# Development of a Prediction Model for Healthy Life Years Without Activity Limitation: National Cross-sectional Study

**DOI:** 10.2196/46634

**Published:** 2023-05-17

**Authors:** Masahiro Nishi, Reo Nagamitsu, Satoaki Matoba

**Affiliations:** 1 Department of Cardiovascular Medicine Graduate School of Medical Science Kyoto Prefectural University of Medicine Kyoto Japan; 2 Department of Health and Welfare Kyoto Prefectural Government Kyoto Japan; 3 Department of Epidemiology for Community Health and Medicine Graduate School of Medical Science Kyoto Prefectural University of Medicine Kyoto Japan

**Keywords:** healthy life years, machine learning, health policy, prediction model, health promotion, activity limitation, cross-sectional study, application tool, life expectancy

## Abstract

**Background:**

In some countries, including Japan—the leading country in terms of longevity, life expectancy has been increasing; meanwhile, healthy life years have not kept pace, necessitating an effective health policy to narrow the gap.

**Objective:**

The aim of this study is to develop a prediction model for healthy life years without activity limitations and deploy the model in a health policy to prolong healthy life years.

**Methods:**

The Comprehensive Survey of Living Conditions, a cross-sectional national survey of Japan, was conducted by the Japanese Ministry of Health, Labour and Welfare in 2013, 2016, and 2019. The data from 1,537,773 responders were used for modelling using machine learning. All participants were randomly split into training (n=1,383,995, 90%,) and test (n=153,778, 10%) subsets. Extreme gradient boosting classifier was implemented. Activity limitations were set as the target. Age, sex, and 40 types of diseases or injuries were included as features. Healthy life years without activity limitations were calculated by incorporating the predicted prevalence rate of activity limitations in a life table. For the wide utility of the model in individuals, we developed an application tool for the model.

**Results:**

In the groups without (n=1,329,901) and with (n=207,872) activity limitations, the median age was 47 (IQR 30-64) and 69 (IQR 54-80) years, respectively (*P*<.001); female sex comprised 51.3% (n=681,794) in the group without activity limitations and 56.9% (n=118,339) in the group with activity limitations (*P*<.001). A total of 42 features were included in the feature set. Age had the highest impact on model accuracy, followed by depression or other mental diseases; back pain; bone fracture; other neurological disorders, pain, or paralysis; stroke, cerebral hemorrhage, or infarction; arthritis; Parkinson disease; dementia; and other injuries or burns. The model exhibited high performance with an area under the receiver operating characteristic curve of 0.846 (95% CI 0.842-0.849) with exact calibration for the average probability and fraction of positives. The prediction results were consistent with the observed values of healthy life years for both sexes in each year (range of difference between predictive and observed values: −0.89 to 0.16 in male and 0.61 to 1.23 in female respondents). We applied the prediction model to a regional health policy to prolong healthy life years by adjusting the representative predictors to a target prevalence rate. Additionally, we presented the health condition without activity limitations index, followed by the application development for individual health promotion.

**Conclusions:**

The prediction model will enable national or regional governments to establish an effective health promotion policy for risk prevention at the population and individual levels to prolong healthy life years. Further investigation is needed to validate the model’s adaptability to various ethnicities and, in particular, to countries where the population exhibits a short life span.

## Introduction

Global public health, secure social systems, and advances in medical practice have contributed to the extension of life expectancy and healthy life years (referred to as the healthy life expectancy) of humans worldwide. With the growing recognition of the importance of taking into account the state of being alive or quality of life, “healthy life years” has come to be focused on as an integrated health indicator that combines not only mortality data but also data on the state of being alive. Healthy life years are not merely defined as life without disability or illness but include a holistic life of well-being. Although life expectancy has been increasing, healthy life years have not yet been kept pace, necessitating an effective health policy to narrow the gap [1,2].

There have been several measures to estimate healthy life years, which are used to evaluate national or regional health status. The World Health Organization has used the health-adjusted life expectancy, a measure of healthy life years based on a specialized health survey producing disability weight on various diseases, injuries, and sequelae [3-5]. In some countries, including Japan—the leading country in terms of longevity, a national survey is conducted to investigate healthy life years using a questionnaire for the presence of activity limitations.

To date, some determinants of healthy life years and the relevant activity limitations have been identified. Typical risk factors, such as obesity, hypertension, hyperglycemia, smoking, and excessive alcohol consumption, are negatively associated with a healthy life [6,7]. Physical activity and exercise [8-11] as well as a diverse healthy diet [12,13] are expected to prolong healthy life years. In addition to fatal diseases, several nonfatal conditions, such as mental health disorders, musculoskeletal problems, and ophthalmic diseases, are also crucial determinants of activity limitations [10,14,15].

Despite the increasing interest in a healthy life for public health campaign and individual health awareness, a prediction model of healthy life years with integrated features has not been reported. In this study, we sought to develop a prediction model for healthy life years without activity limitations using machine learning and to deploy the model to a health policy in prolonging healthy life years at the population and individual levels.

## Methods

### Data Description

The Comprehensive Survey of Living Conditions, a cross-sectional national survey, is conducted every 3 years by the Japanese Ministry of Health, Labour and Welfare to investigate the fundamental aspects of the nation’s livelihood, such as health, medical care, welfare, pension, and income [16]. In health questionnaire of the Comprehensive Survey of Living Conditions, subjective symptoms, health problems in daily life, disease or injury under treatment, subjective health assessment, worries and stress, mental state, and receiving rate of health check-ups are surveyed. The response rate in 2019 was 72.5% according to the Japanese Ministry of Health, Labour and Welfare. Among the data in 2013, 2016, and 2019, data from 1,537,773 responders were used for the analysis. The activity limitations of responders were evaluated using responses to the questions, “Do you have any health problem which limits your daily activity?” Respondents who answered “yes” were categorized as the “activity limitations” group, and those who answered “no” were categorized into the “no activity limitation” group. Activity limitations, age, sex, and the 40 types of diseases or injuries under treatment were included in the analysis.

### Model Description

The activity limitations, which were classified as binary, were set as model target; the “activity limitations” group was classified as 1, and the “no activity limitation” group as 0. Age, sex, and the 40 types of diseases or injuries under treatment were included as features. We implemented the extreme gradient boosting (XGB) classifier—a widely used supervised tree-based model, which uses labeled data sets to train a model [17-19]—for the binary classification of activity limitations using the scikit-learn 1.1.1 package [20] in Python 3.10.4. Using the “*train_test_split*” function, we randomly split the data set into training (n=1,383,995, 90%) and test (n=153,778, 10%) subsets [21,22]. We selected the best feature set using the recursive feature elimination function in the training data set, with the XGB classifier as an estimator. To determine the best hyperparameter values providing the highest model performance, we used *the GridSearchCV* function for training subset with the five-fold cross validation. A total of 480 XGB models were compared using different combinations of hyperparameters. Consequently, we selected a model with hyperparameters (n_estimators=200, max_depth=9, eta=0.1, min_child_weight=2, max_delta_step=5, and subsample=0.5) yielding the highest area under the receiver operating characteristic curve (AUROC). Finally, the model performance was evaluated for the test subsets.

The impact of the features on the model accuracy was estimated by permutation importance, which is defined as difference of error when a feature value is randomly shuffled, assigning 1.0 to the highest impact. The SHapley Additive exPlanations (SHAP) value, which explains a feature contribution on model output in each sample, was used to evaluate the effect of features on the model output [23]. We calculated the area under the curve and log loss, a measure of how close predictive probability is to observed value, as the model accuracy metrics for the XGB classifier, random forest, and logistic regression. Other metrics were calculated based on the confusion matrix. We used *calibration_curve* function for the model calibration between predictive probability and fraction of positives, dividing samples into 10 bins according to predictive probability. Cost-benefit was calculated to determine the optimal cut-off of prediction by the sum of true positive and true negative as 0, false positive as +1, and false negative as −1 in the confusion matrix. Healthy life years without activity limitations of female respondents in Kyoto Prefecture were predicted using the prediction model with the original and target prevalence rates of the representative diseases, which are much higher than the mean prevalence rates in the whole country. Random sampling was performed for populations with each disease to achieve target prevalence rate. The web-based application tool for the model was developed on a web application platform based on the programming code [24].

### Statistical Analysis

General descriptive statistics were performed in R (version 4.2.0; R Core Team) [25]. Categorical values are represented as numbers (along with percentages), and numerical values are represented as medians (IQRs). The chi-square test was used for categorical values, and the Mann-Whitney *U* test was used for continuous values with a nonparametric distribution. *P*<.01 was considered statistically significant. The health condition without activity limitation (HCAL) index was calculated by subtracting the percentage of predictive probability for activity limitations from 100. Curve fitting was performed using third-order polynomial regression. Healthy life years without activity limitation were calculated using Sullivan’s method, which is widely used to calculate life expectancy based on age-stratified mortality rate and life table, incorporating the prevalence rate of activity limitations to a life table in Japan [26,27].

### Ethical Considerations

The study was approved by the ethics committee of Kyoto Prefectural University of Medicine (approval number ERB-C-2496). This study conformed to the principles outlined in the Declaration of Helsinki. Since this study used only existing national survey data, opt-out decline was adopted for participants on the university website instead of informed consent. The study data are anonymous. There was no compensation for participants.

## Results

### Participant Characteristics

The characteristics of participants (N=1,537,773) are described and stratified according to the presence of activity limitations (Table 1). In the groups without (n=1,329,901) and with activity limitations (n=207,872), the median age was 47 (IQR 30-64) and 69 (IQR 54-80) years, respectively (*P*<.001), and female sex comprised 51.3% (n=681,79) of the participants in the group without activity limitations (vs n=118,339, 56.9%; *P*<.001). Diseases under treatment, except for infertility, were also significantly different between the two groups (depression or other mental disease: n=13,727, 1% vs n=15,347, 7.4%; dementia: n=2420, 0.2% vs n=8667, 4.2%; stroke, cerebral hemorrhage, or infarction: n=8452, 0.6% vs n=10,818, 5.2%; angina or myocardial infarction: n=16,467, 1.2% vs n=13,043, 6.3%; rheumatoid arthritis: n=5153, 0.4% vs n=6239, 3%; arthritis: n=15,682, 1.2% vs n=19,753, 9.5%; back pain: n=42,856, 3.2% vs n=37,980, 18.3%; kidney disease: n=7415, 0.6% vs n=7866, 3.8%; malignant neoplasm or cancer: n=7594, 0.6% vs n=6249, 3%). All participants (N=1,537,773) were randomly split into training (n=1,383,995, 90%) and test (n=153,778, 10%) subsets with similar characteristics (Table S1 in Multimedia Appendix 1).

**Table 1:** Characteristics of participants stratified by presence of activity limitations.

Characteristics	All (N=1,537,773)	Without activity limitation (n=1,329,901)	With activity limitation (n=207,872)	*P* value
Age (years), median (IQR)	50 (32-67)	47 (30-64)	69 (54-80)	<.001
Sex (female), n (%)	800,133 (52)	681,794 (51.3)	118,339 (56.9)	<.001
Diabetes, n (%)	77,672 (5.1)	53,671 (4)	24,001 (11.5)	<.001
Thyroid disease, n (%)	19,811 (1.3)	14,360 (1.1)	5451 (2.6)	<.001
Depression or other mental disease, n (%)	29,074 (1.9)	13,727 (1)	15,347 (7.4)	<.001
Dementia, n (%)	11,087 (0.7)	2420 (0.2)	8667 (4.2)	<.001
Parkinson disease, n (%)	3194 (0.2)	533 (0)	2661 (1.3)	<.001
Other neurological disorders, pain, or paralysis, n (%)	11,028 (0.7)	4311 (0.3)	6717 (3.2)	<.001
Eye disease, n (%)	83,577 (5.4)	52,941 (4)	30,636 (14.7)	<.001
Ear disease, n (%)	16,411 (1.1)	9354 (0.7)	7057 (3.4)	<.001
Stroke, cerebral hemorrhage, or infarction, n (%)	19,270 (1.3)	8452 (0.6)	10,818 (5.2)	<.001
Angina and myocardial infarction, n (%)	29,510 (1.9)	16,467 (1.2)	13,043 (6.3)	<.001
Other cardiovascular disease, n (%)	28,703 (1.9)	15,653 (1.2)	13,050 (6.3)	<.001
Acute nasopharyngitis and common cold, n (%)	5125 (0.3)	3549 (0.3)	1576 (0.8)	<.001
Infertility, n (%)	1536 (0.1)	1359 (0.1)	177 (0.1)	.02
Dental disease, n (%)	80,560 (5.2)	63,668 (4.8)	16,892 (8.1)	<.001
Gout, n (%)	15,396 (1)	12,090 (0.9)	3306 (1.6)	<.001
Obesity, n (%)	8038 (0.5)	5013 (0.4)	3025 (1.5)	<.001
Dyslipidemia, n (%)	81,338 (5.3)	63,404 (4.8)	17,934 (8.6)	<.001
Hypertension, n (%)	206,103 (13.4)	153,500 (11.5)	52,603 (25.3)	<.001
Allergic rhinitis, n (%)	32,310 (2.1)	24,472 (1.8)	7838 (3.8)	<.001
Chronic obstructive pulmonary disease, n (%)	2250 (0.1)	813 (0.1)	1437 (0.7)	<.001
Asthma, n (%)	19,022 (1.2)	13,149 (1)	5873 (2.8)	<.001
Other respiratory disease, n (%)	15,134 (1)	8517 (0.6)	6617 (3.2)	<.001
Stomach or duodenum disease, n (%)	26,285 (1.7)	17,048 (1.3)	9237 (4.4)	<.001
Liver or gallbladder disease, n (%)	14,624 (1)	9283 (0.7)	5341 (2.6)	<.001
Other digestive disease, n (%)	18,656 (1.2)	11,028 (0.8)	7628 (3.7)	<.001
Atopic dermatitis, n (%)	14,353 (0.9)	11,553 (0.9)	2800 (1.3)	<.001
Other skin disease, n (%)	29,205 (1.9)	20,475 (1.5)	8730 (4.2)	<.001
Rheumatoid arthritis, n (%)	11,392 (0.7)	5153 (0.4)	6239 (3)	<.001
Arthritis, n (%)	35,435 (2.3)	15,682 (1.2)	19,753 (9.5)	<.001
Stiff shoulder, n (%)	43,474 (2.8)	28,093 (2.1)	15,381 (7.4)	<.001
Back pain, n (%)	80,836 (5.3)	42,856 (3.2)	37,980 (18.3)	<.001
Osteoporosis, n (%)	28,790 (1.9)	14,606 (1.1)	14,184 (6.8)	<.001
Kidney disease, n (%)	15,281 (1)	7415 (0.6)	7866 (3.8)	<.001
Prostatic hypertrophy, n (%)	19,932 (1.3)	12,293 (0.9)	7639 (3.7)	<.001
Menopausal or postmenopausal disorder, n (%)	3041 (0.2)	1980 (0.1)	1061 (0.5)	<.001
Bone fracture, n (%)	10,464 (0.7)	3345 (0.3)	7119 (3.4)	<.001
Other injury or burns, n (%)	10,230 (0.7)	5451 (0.4)	4779 (2.3)	<.001
Anemia or blood disease, n (%)	10,660 (0.7)	5980 (0.4)	4680 (2.3)	<.001
Malignant neoplasm or cancer, n (%)	13,843 (0.9)	7594 (0.6)	6249 (3)	<.001
Pregnancy, puerperium, threatened abortion, or placenta previa, n (%)	2198 (0.1)	1565 (0.1)	633 (0.3)	<.001

### Model Performance Evaluation

**Figure 1:**
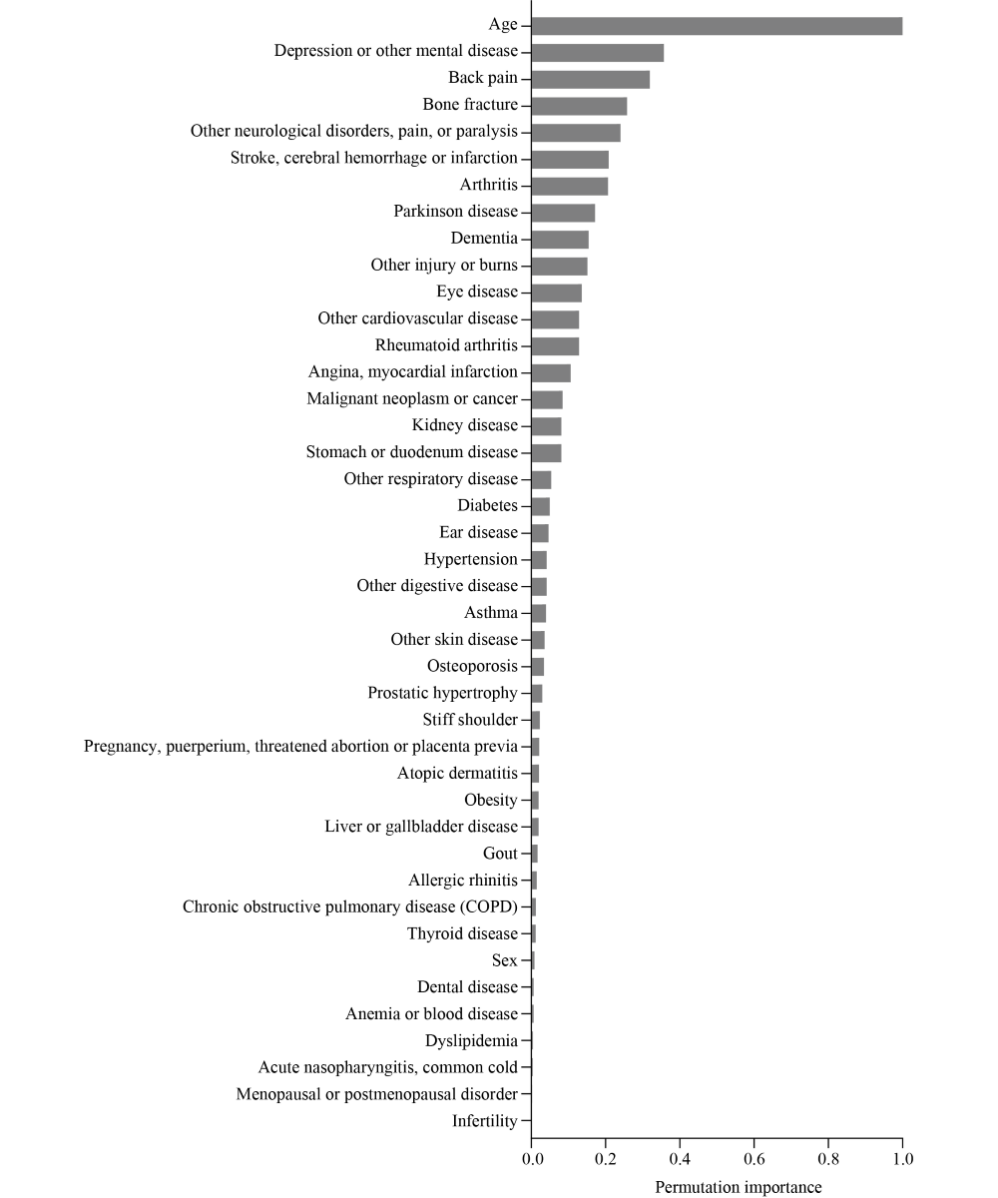
Feature impact estimated by permutation importance. Permutation importance was calculated for features using test data.

To create the model feature set, the AUROC was compared for each feature number. A total of 42 features were included in the feature set because they had the highest AUROC (Figure S1 in Multimedia Appendix 1). The feature impact estimated by permutation importance showed that age had the highest impact on model accuracy, followed by depression or other mental diseases; back pain; bone fracture; other neurological disorders, pain, or paralysis; stroke, cerebral hemorrhage, or infarction; arthritis; Parkinson disease; dementia; and other injuries or burns (Figure 1).

**Figure 2:**
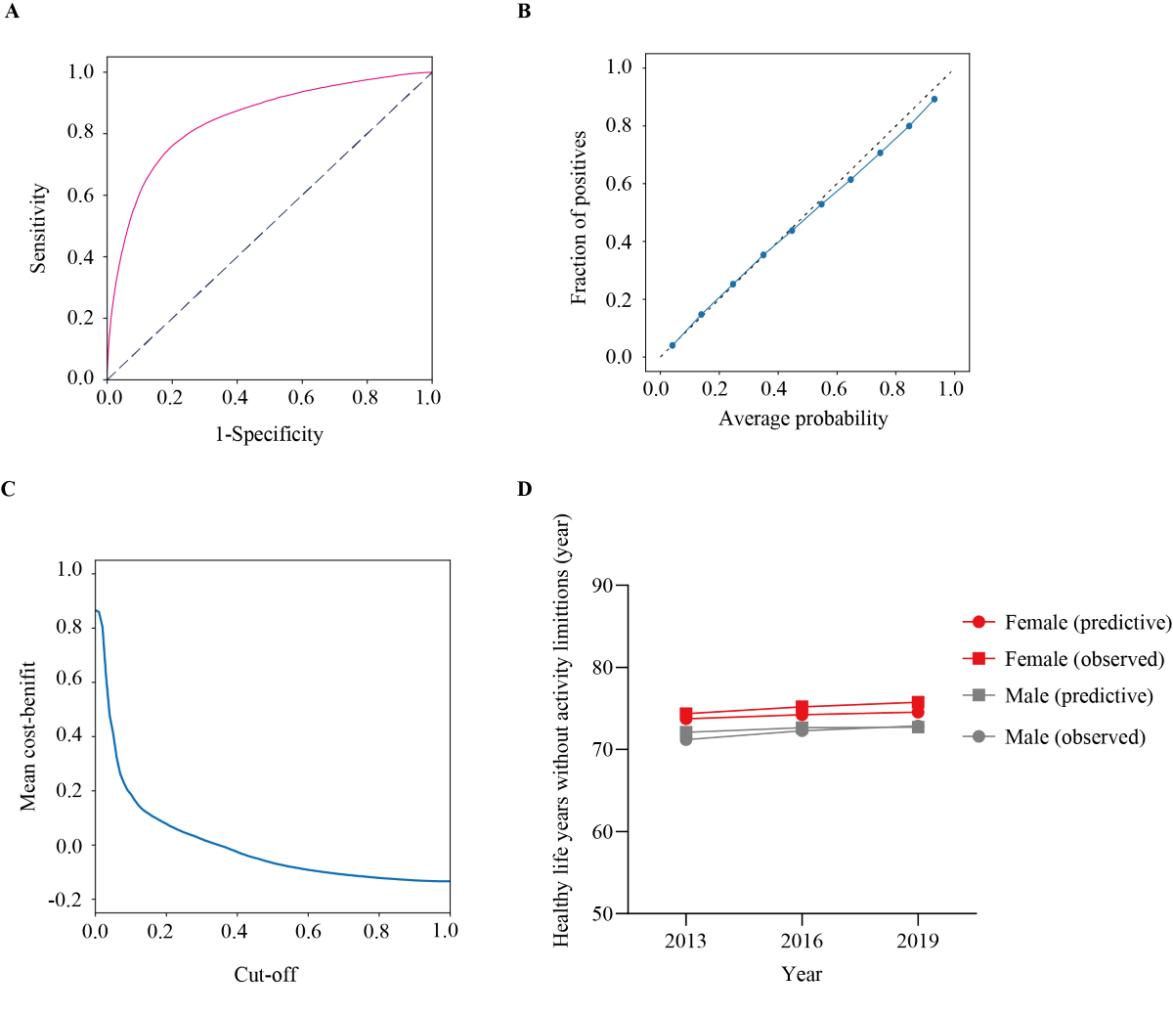
Evaluation of model performance. (A) Receiver operating characteristic curve for the model. Area under the receiver operating characteristic curve was 0.846 (95% CI 0.842-0.849). (B) Calibration plot for the model; samples were divided into 10 bins according to probability. (C) Mean cost-benefit curve. (D) Predictive and observed value of healthy life years for male and female respondents in each year.

The accuracy metrics were compared for some learners. We selected the XGB classifier as a learner because it exhibited a high AUORC and low log loss compared with random forest and logistic regression (Table S2 in Multimedia Appendix 1). The model performance was evaluated by depicting the receiver operating characteristic curve, and the AUROC was 0.846 (95% CI 0.842-0.849; Figure 2A). The calibration plot exhibited exact calibration for the average probability and fraction of positives (Figure 2B). We set the cut-off of 0.31 according to the lowest absolute value of the mean cost-benefit (Figure 2C). The prediction results were consistent with the observed values of healthy life years for both sexes in each year (range of difference between predictive and observed value: −0.89 to 0.16 in male and 0.61 to 1.23 in female respondents; Figure 2D). Thus, the developed model exhibited a markedly high performance in predicting healthy life years without activity limitations.

### Model Application for Population and Individual Health

For model application at the population level, we used the prediction model for a regional health policy regarding healthy life years (Table S3 in Multimedia Appendix 1). Healthy life years without activity limitations of females in Kyoto prefecture in Japan were predicted using the prediction model with the original and target prevalence rates of representative predictors, such as depression or other mental diseases, back pain, and stiff shoulder. As a result, healthy life years without activity limitations were simulated to be prolonged from 73.25 in the original to 73.81 in the target, a difference of 0.56. Herein, we demonstrate the use of a prediction model for a regional health policy to prolong healthy life years at the population level.

**Figure 3:**
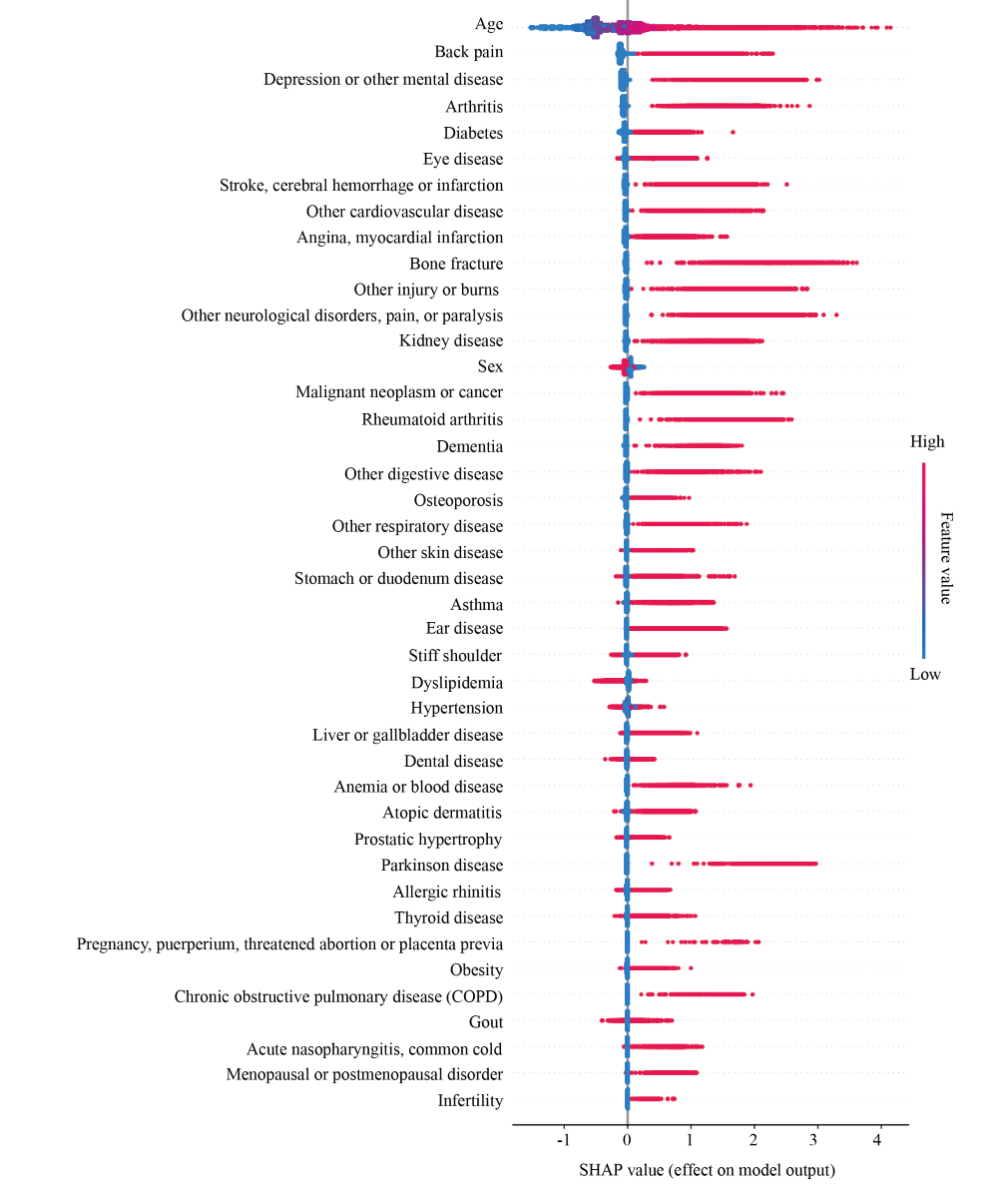
Feature effect on model output. SHapley Additive exPlanations (SHAP) value was calculated for features using test data.

**Figure 4:**
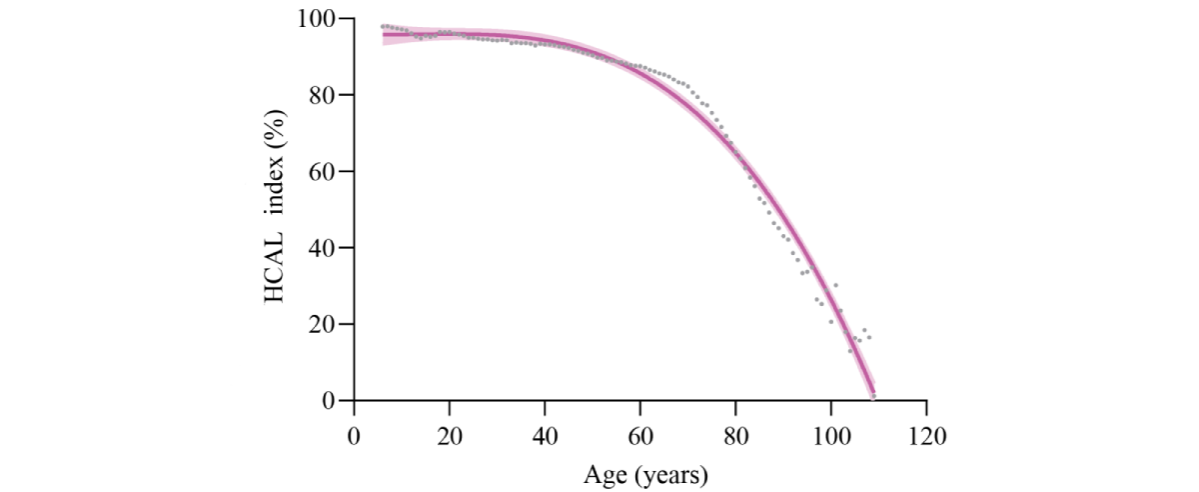
Health condition without activity limitation (HCAL) per age. The HCAL index indicates subtraction of the percentage of predictive probability from 100. Curve fitting was done using third-order polynomial regression. Error bar indicates 95% CI. Each dot represents mean HCAL index per age.

To enhance the interpretability of the feature effect on the model output, the SHAP value is displayed for each feature (Figure 3). This shows that age has the greatest effect on model output. HCAL index was decreased by aging (Figure 4). For the wide utility of the model in individuals, we developed a web-based application tool to display the HCAL index (Figure S2 in Multimedia Appendix 1).

## Discussion

### Principal Findings

In this study, we developed a prediction model for healthy life years without activity limitations using machine learning by analyzing a cross-sectional national survey. The model exhibited markedly high performance with a high AUROC and subtle differences between the observed and predicted values of healthy life years without activity limitations. We applied the prediction model to a regional health policy to prolong healthy life years by adjusting the representative predictors to a target prevalence rate. Additionally, we presented the HCAL index, followed by the application development, for individual health promotion.

We estimated the feature impact on model accuracy by permutation importance and the effect on model output by the SHAP value. The impact of features on model accuracy showed that age had the highest impact, followed by depression or other mental disease; back pain; bone fracture; other neurological disorders, pain, or paralysis; stroke, cerebral hemorrhage, or infarction; arthritis; Parkinson disease; dementia; other injuries or burns. Interestingly, the high-impact features included several nonfatal conditions, such as mental disorders, musculoskeletal problems, and neurological diseases. Our findings were consistent with previous reports that suggest mental health disorders and musculoskeletal problems are crucial predictive factors for activity limitations [10,15]. Of mental disorders, schizophrenia and major depression have heavy disability weight according to the Global Burden of Disease Study [3]. A study using data from the Global Burden of Disease Study found no association between health output and common health system per prefecture in Japan [28]. These findings indicate that healthy life years without activity limitations largely rely on mental, musculoskeletal, or neurological causes rather than other typical lifestyle-related factors.

We leveraged machine learning to predict healthy life years without activity limitations. The presence of activity limitations assessed by a subjective questionnaire was used for the model target; nonetheless, healthy life years could be predicted accurately with the objective 42 features using machine learning. Machine learning facilitated model deployment by application development at the population and individual levels. Natural language processing has been applied to calculate the health-adjusted life expectancy using electronic medical records [29]. Machine learning combined with natural language processing for electronic medical records will provide a solution for global health issues regarding healthy life years.

We demonstrated the model application for population and individual health. Healthy life years without activity limitations of females in Kyoto prefecture were simulated using the prediction model with the original and target prevalence rates of representative predictors. Thus, the model could be used to present effective ways to prolong healthy life years for a regional health policy. Moreover, the application tool was developed using the model for wide utility in individual health promotion. The tool can be used in several situations, such as health check, patient education, and outpatient clinics. Our model was developed with machine learning and can be used for prediction of population-level healthy life years as well as individual health conditions, increasing its feasibility compared with other measures for healthy life years.

### Limitations

This study had certain limitations, as it was based on a survey that included subjective data, and only data from Japan were used. Further investigation is needed to validate the model’s adaptability to various ethnicities and, in particular, countries where the population has a short life span. For complexity of machine learning to explain and interpret, we used permutation importance and SHAP values for feature impact.

### Conclusions

In conclusion, we developed a prediction model for healthy life years without activity limitations, using machine learning. The prediction model will enable the national or regional government to establish an effective health promotion policy for risk prevention at the population and individual levels to prolong healthy life years. It would be interesting to investigate the model’s applicability to other countries and ethnicities.

## Appendix

Multimedia Appendix 1 Supplementary materials.

## Abbreviations

AUROC: area under the receiver operating characteristic curve
HCAL: health condition without activity limitation
SHAP: SHapley Additive exPlanations
XGB: extreme gradient boosting

## Data Availability

The data set generated and analyzed during this study is not publicly available due to restrictions imposed by Japanese Ministry of Health, Labour and Welfare (the data provider) but can be obtained by contacting the ministry based on reasonable request.
