# Supplementary material for: Development of a Prediction Model for Healthy Life Years Without Activity Limitation: National Cross-sectional Study
Source: JMIR Public Health Surveill. 2023 May 17;9:e46634. doi: 10.2196/46634 (PMC10233441; doi:10.2196/46634)
Supplement: Multimedia Appendix 1 [file publichealth_v9i1e46634_app1.pdf]

Characteristics	Training data (n = 1,383,995, 90%)	Test data (n = 153,778, 10%)	*P* value
Age	50 (32-67)	50 (32-67)	.08
Sex (female)	663,825/1,383,995 (48)	73,815/153,778 (48)	.78
Activity limitation	187,305/1,383,995 (13.5)	20,567/153,778 (13.4)	.08
Diabetes	69,804/1,383,995 (5)	7,868/153,778 (5.1)	.21
Thyroid disease	17,782/1,383,995 (1.3)	2,029/153,778 (1.3)	.25
Depression or other mental disease	26,132/1,383,995 (1.9)	2,942/153,778 (1.9)	.50
Dementia	9,966/1,383,995 (0.7)	1,121/153,778 (0.7)	.70
Parkinson disease	2,887/1,383,995 (0.2)	307/153,778 (0.2)	.48
Other neurological disorders, pain, or paralysis	9,897/1,383,995 (0.7)	1,131/153,778 (0.7)	.37
Eye disease	75,026/1,383,995 (5.4)	8,551/153,778 (5.6)	.02
Ear disease	14,802/1,383,995 (1.1)	1,609/153,778 (1)	.40
Stroke, cerebral hemmorrhage or infarction	17,292/1,383,995 (1.2)	1,978/153,778 (1.3)	.22
Angina, myocardial infarction	26,519/1,383,995 (1.9)	2,991/153,778 (1.9)	.43
Other cardiovascular disease	25,865/1,383,995 (1.9)	2,838/153,778 (1.8)	.52
Acute nasopharyngitis, common cold	4,616/1,383,995 (0.3)	509/153,778 (0.3)	.88
Infertility	1,386/1,383,995 (0.1)	150/153,778 (0.1)	.79
Dental disease	72,460/1,383,995 (5.2)	8,100/153,778 (5.3)	.60
Gout	13,862/1,383,995 (1)	1,534/153,778 (1)	.89
Obesity	7,267/1,383,995 (0.5)	771/153,778 (0.5)	.22
Dyslipidemia	73,382/1,383,995 (5.3)	7,956/153,778 (5.2)	.03
Hypertension	185,542/1,383,995 (13.4)	20,561/153,778 (13.4)	.69
Allergic rhinitis	29,035/1,383,995 (2.1)	3,275/153,778 (2.1)	.41
Chronic obstructive pulmonary disease (COPD)	2,036/1,383,995 (0.1)	214/153,778 (0.1)	.46
Asthma	17,051/1,383,995 (1.2)	1,971/153,778 (1.3)	.09
Other respiratory disease	13,657/1,383,995 (1)	1,477/153,778 (1)	.32
Stomach or duodenum disease	23,618/1,383,995 (1.7)	2,667/153,778 (1.7)	.43
Liver or gallbladder disease	13,123/1,383,995 (0.9)	1,501/153,778 (1)	.29
Other digestive disease	16,760/1,383,995 (1.2)	1,896/153,778 (1.2)	.46
Atopic dermatitis	12,882/1,383,995 (0.9)	1,471/153,778 (1)	.32
Other skin disease	26,191/1,383,995 (1.9)	3,014/153,778 (2)	.06
Rheumatoid arthritis	10,292/1,383,995 (0.7)	1,100/153,778 (0.7)	.22
Arthritis	31,918/1,383,995 (2.3)	3,517/153,778 (2.3)	.64
Stiff shoulder	39,074/1,383,995 (2.8)	4,400/153,778 (2.9)	.39
Back pain	72,701/1,383,995 (5.3)	8,135/153,778 (5.3)	.54
Osteoporosis	25,929/1,383,995 (1.9)	2,861/153,778 (1.9)	.72
Kidney disease	13,727/1,383,995 (1)	1,554/153,778 (1)	.49
Prostatic hypertrophy	17,947/1,383,995 (1.3)	1,985/153,778 (1.3)	.85
Menopausal or postmenopausal disorder	2,739/1,383,995 (0.2)	302/153,778 (0.2)	.92
Bone fracture	9,445/1,383,995 (0.7)	1,019/153,778 (0.7)	.37
Other injury or burning	9,182/1,383,995 (0.7)	1,048/153,778 (0.7)	.41
Anemia or blood disease	9,614/1,383,995 (0.7)	1,046/153,778 (0.7)	.52
Malignant neoplasm or cancer	12,424/1,383,995 (0.9)	1,419/153,778 (0.9)	.33
Pregnancy, puerperium, threatened abortion or placenta previa	1,998/1,383,995 (0.1)	200/153,778 (0.1)	.16

Table S1. Characteristics of participants in training and test data.

Model	AUC (95% CI)	Log loss (95% CI)
XGB classifier	0.85 (0.84–0.85)	0.29 (0.28–0.29)
Random forest	0.84 (0.84–0.85)	0.29 (0.28–0.29)
Logistic regression	0.84 (0.84–0.84)	0.50 (0.50–0.50)

Table S2. Performance of machine-learning models. Model performance was evaluated by the AUC and log loss for the XGB classifier, random forest, and logistic regression using the test data. AUC: area under the curve; CI: confidence interval.

	Original	Target	Difference
**Depression or other mental disease (%)**	2.76	2.23	−0.53
**Back paine (%)**	4.61	3.59	−1.02
**Stiff shoulder (%)**	6.65	5.77	−0.88
**Healthy life years (years)**	73.25	73.81	0.56

Table S3. Use case of the prediction model for healthy life years. Healthy life years without activity limitations of female respondents in Kyoto Prefecture was predicted using the prediction model with the original and target prevalence of the representative predictors. Random sampling was performed for the participants with each disease under treatment to achieve target prevalence.

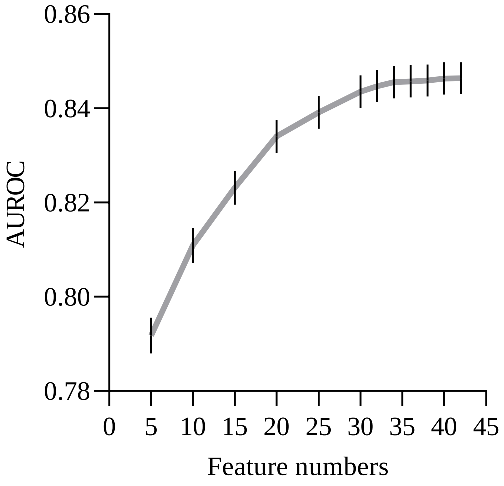

Figure S1. Feature selection and impact. Area under the ROC curve (AUROC) for each feature number is shown. Error bar indicates 95% confidence interval. ROC: receiver operating characteristic.

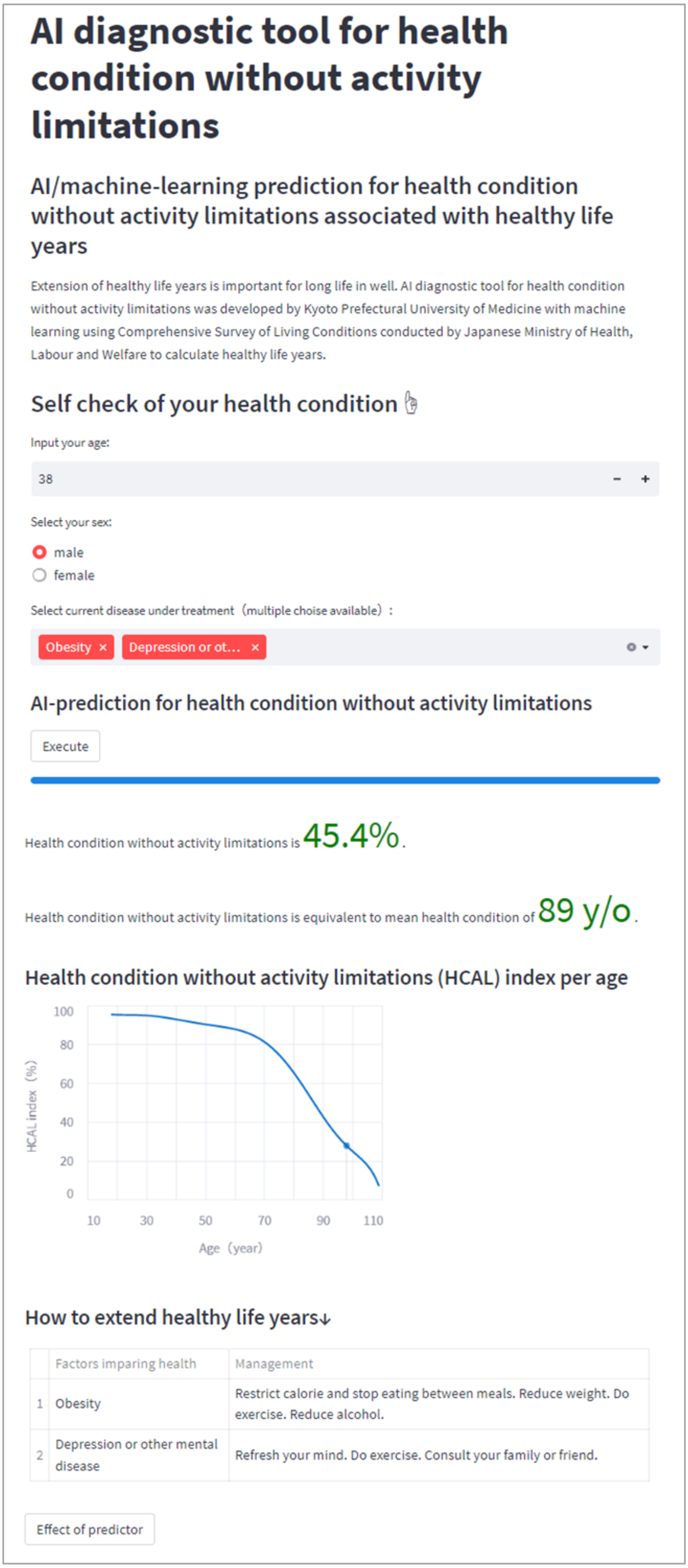

Figure S2. Image of AI diagnostic web tool for health condition. For wide utility of the model in individuals, we developed an online application tool to display the health condition without activity limitation (https://self-check-health.herokuapp.com/).
